# Integration Host Factor Binds DNA Holliday Junctions

**DOI:** 10.3390/ijms24010580

**Published:** 2022-12-29

**Authors:** Shawn H. Lin, Dacheng Zhao, Vivian Deng, Veronica K. Birdsall, Suzanne Ho, Olga Buzovetsky, Candice M. Etson, Ishita Mukerji

**Affiliations:** 1 Molecular Biology and Biochemistry Department, Wesleyan University, Middletown, CT 06459, USA; 2 Molecular Biophysics Program, Wesleyan University, Middletown, CT 06459, USA

**Keywords:** integration host factor, Holliday Junction, single-molecule FRET, fluorescence

## Abstract

Integration host factor (IHF) is a nucleoid-associated protein involved in DNA packaging, integration of viral DNA and recombination. IHF binds with nanomolar affinity to duplex DNA containing a 13 bp consensus sequence, inducing a bend of ~160° upon binding. We determined that IHF binds to DNA Four-way or Holliday junctions (HJ) with high affinity regardless of the presence of the consensus sequence, signifying a structure-based mechanism of recognition. Junctions, important intermediates in DNA repair and homologous recombination, are dynamic and can adopt either an open or stacked conformation, where the open conformation facilitates branch migration and strand exchange. Using ensemble and single molecule Förster resonance energy transfer (FRET) methods, we investigated IHF-induced changes in the population distribution of junction conformations and determined that IHF binding shifts the population to the open conformation. Further analysis of smFRET dynamics revealed that even in the presence of protein, the junctions remain dynamic as fast transitions are observed for the protein-bound open state. Protein binding alters junction conformational dynamics, as cross correlation analyses reveal the protein slows the transition rate at 1 mM Mg^2+^ but accelerates the transition rate at 10 mM Mg^2+^. Stopped flow kinetic experiments provide evidence for two binding steps, a rapid, initial binding step followed by a slower step potentially associated with a conformational change. These measurements also confirm that the protein remains bound to the junction during the conformer transitions and further suggest that the protein forms a partially dissociated state that allows junction arms to be dynamic. These findings, which demonstrate that IHF binds HJs with high affinity and stabilizes junctions in the open conformation, suggest that IHF may play multiple roles in the processes of integration and recombination in addition to stabilizing bacterial biofilms.

## 1. Introduction

Integration Host Factor (IHF) is a small, heterodimeric, nucleoid associated protein that is involved in a number of cellular processes, including transcription, recombination, replication and viral integration. Originally identified in integration of λ-DNA, IHF has also been shown to be important for the Cas1-Cas2 integrase, where IHF-induced distortion of the leader sequence improves specificity and efficiency of integration [1,2].

The protein binds with high affinity to DNA duplexes containing its 13 bp consensus sequence, WATCARNNNNTTR (W is A or T, R is A or G, N is any nucleotide). Upon binding IHF induces a pronounced bend in the DNA [3], which was found to be almost 160° in the X-ray IHF-DNA co-crystal structure [4,5]. When binding to its consensus sequence IHF employs both direct and indirect readout mechanisms for recognition. In the co-crystal structure, IHF can be seen to wrap around the DNA with two β-strand arms extending from an α-helical body. Two proline residues at the tips of the arms insert into the DNA, kinking the DNA. An A-tract sequence located upstream of the consensus sequence interacts with the α-helical body on the right-hand side of the protein, while the consensus sequence (shown in magenta) interacts with the arms and the left-hand side of the α-helical body [4].

Proteins in the DNABII family, which contains IHF and structural homologue HU, are known as architectural proteins as they recognize distorted DNA substrates with high affinity and also bend DNA. These proteins have also been shown to bind and compact DNA, potentially regulating gene expression through supercoiling and modulation of genome architecture [6,7]. Although HU and IHF are structural homologues; they differ in their DNA binding properties, as HU is a non-sequence-specific DNA binding protein [8,9]. Both proteins have been shown to be critical components of bacterial biofilms, which contain extracellular DNA (eDNA) as a matrix component [10,11]. The branched matrix of the eDNA contains Holliday Junction-like structures, which IHF and HU bind and stabilize [12]. HU has previously been shown to bind to Holliday Junctions [13,14,15]; however, direct evidence of IHF binding to such structures is lacking.

Holliday Junctions are known intermediates in DNA repair and recombination processes [16,17] and have been recently shown to be structural components of pathogenic biofilms [12]. Junctions are flexible, dynamic structures that can exist in a variety of conformations [16,18,19]. X-ray crystal structures have demonstrated that junctions consist of four DNA strands with a central region where the strands cross and interchange [20,21]. Due to the high amounts of negative charge in the central region, ions have been structurally observed in that region [22,23]. Relatively high ion concentrations are required for the junction to adopt a stacked structure, where the arms are coaxially stacked on one another and are oriented in an anti-parallel manner [20,24]. In the open structure, the junction can branch migrate, while the stacked structure is often a target for resolvases and endonucleases [25,26,27].

The junction, J3, has been extensively characterized by Lilley, Ha and co-workers and is used as a model system in our studies [28,29]. At physiological concentrations of ions, this matched, non-migrating junction is thermodynamically stable [30] and preferentially adopts the stacked configuration. Time-resolved and single molecule FRET experiments have shown that the junction prefers one isomer conformation at a ratio of 80:20 [29,31]. Previously, we examined HU binding to a J3 model junction and observed that the protein bound and stabilized the stacked configuration with high affinity [15].

Given recent reports regarding IHF participation in bacterial biofilms containing HJ [12] and IHF promotion of homologous recombination in *P. putida* [32], we elected to investigate IHF binding to DNA four-way junctions at a molecular level. In this study we have examined the binding of IHF to junctions as a function of arm length and in the presence of the consensus sequence using gel and fluorescence binding assays. We find that IHF binds all junctions with high affinity, suggesting that the protein binds to the central region of the junction. Steady state, time-resolved and single molecule FRET experiments demonstrate that IHF binds to open junctions with high affinity and stabilizes them in that conformation. The smFRET measurements, which have been previously shown to effectively monitor junction kinetics [29,33] demonstrate that the IHF bound state is conformationally dynamic suggesting formation of a partially dissociated state that does not constrain junction arm motion.

Previous studies have determined that IHF binding to duplex DNA exhibits biphasic binding kinetics where the fast phase is a non-sequence specific interaction in which the protein searches for its specific site through facilitated diffusion. The slower phase is associated with DNA bending and corresponds to specific recognition of DNA substrates [34,35,36]. Measurement of IHF-HJ binding kinetics indicate that the mechanism of binding is similar, an initial non-specific binding interaction followed by specific binding. smFRET measurements of the conformer transition rate confirm that the rate constants for binding and conformer transition are of the same magnitude (20 s^−1^ vs. 80 s^−1^, respectively), supporting a model in which the protein specifically binds open junction in a conformational capture mechanism similar to that reported for RuvC [37].

## 2. Results

### 2.1. IHF Binds to DNA Holliday or Four Way Junctions

**Figure 1:**
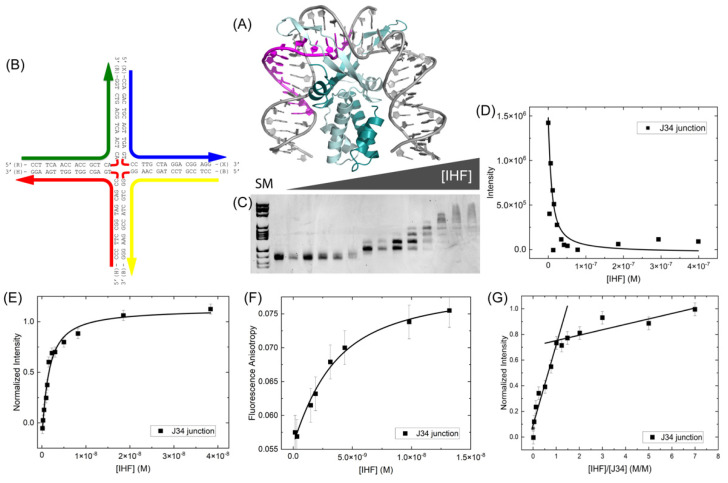
(**A**) Cartoon representation of the IHF protein bound to the H’ consensus site shown in magenta (PDBID: 1IHF) (**B**) Schematic of the J34 junction with sequence (**C**) EMSA of IHF binding to 5 nM J34 junction. SM indicates size marker and is in lane 1, followed by free DNA. IHF concentration ranged from 0–400 nM in the lanes were the following concentrations: 3.2, 6.4, 11.9, 15.2, 23.3, 34.5, 41.9, 51.0, 78.2, 132, 186, 293, 399 nM. (**D**) A 1:1 binding analysis of the free DNA yields an apparent *K*_D_ of 58 ± 36 nM. (**E**) Fluorescence intensity measurements performed with a 6-MI labeled junction at a constant concentration of 0.5 nM, yield a *K*_D_ value ≤ 2.2 nM. (**F**) Fluorescence anisotropy measurements performed with a fluorescein end-labeled J34 junction at a 2 nM concentration yielded a *K*_D_ value of 2 nM. (**G**) Stoichiometry measurements were performed by fluorescence intensity with 20 nM of a fluorescein end-labeled J34 junction. The break point in the slopes occurs at 20 nM IHF consistent with a 1:1 binding stoichiometry. All measurements were performed in the presence of 70 mM KCl, 5 mM Tris, pH 7.6, 0.5% ethylene glycol. Error bars represent the SD from at least 3 experiments.

We have characterized the binding of the *E. coli* Integration Host Factor (IHF) protein (Figure 1A) with three different four-way or Holliday junctions (HJ). The junctions used in our study are based on the well characterized junction J3 [28,29,31], are perfectly matched in sequence and are immobile (Figure 1). Binding measurements were done in the gel, using the method of electrophoretic mobility shift assay (EMSA) and in solution, using fluorescence intensity and anisotropy measurements. We have developed a model HJ based on the J3 junction but with four 17 bp arms (referred to as J34) (Figure 1B). Using EMSA, we measure an equilibrium dissociation constant or *K*_D_ of 58 ± 36 nM for IHF binding to this junction by analyzing the free or unbound DNA (Figure 1C,D). This is comparable to what we and others have observed previously for IHF binding to a 34 bp duplex containing the H1 consensus sequence [38,39] and suggests that IHF exhibits similar binding affinity for HJ or four-way junction structures.

To further explore the relative affinity of IHF for the HJ, we examined the binding using fluorescence spectroscopy (Figure 1E,F). These fluorescence binding experiments were done either with an end-labeled probe or by incorporating the fluorescent nucleoside analog 6-methylisoxanthopterin (6-MI) [40]. The placement of the probes or the probes themselves did not affect our findings, as both fluorophores yielded similar solution *K*_D_ values of ≤3 nM. Our previous in solution measurements with a 34-mer duplex containing the H1 sequence yielded *K*_D_ values ≤4 nM when measuring the affinity by fluorescence anisotropy, confirming that the relative affinity IHF exhibits for this junction is comparable to that exhibited for duplexes containing the consensus sequence [38,41].

Using fluorescence intensity, we monitored the stoichiometry of protein binding and despite the observance of multiple bands in the mobility shift assay, we detect a 1:1 binding stoichiometry as shown in Figure 1G. Conformational changes of the junction, which affect the relative mobility, likely lead to the observance of multiple bands in the gel [28,42]. In the solution stoichiometric analysis, the concentration of junction is ten times higher than the *K*_D_ to ensure that all protein introduced is bound to the junction. As shown, the intensity increases with added protein until all binding sites are filled, at which point further addition of protein does not typically lead to binding or increased intensity. In this case, a shallow intensity increase is observed in the plateau region, which is attributed to non-specific binding of the protein to the junction. We expect that this non-specific binding occurs on the arms of the junction as discussed below. The break point reflecting the concentration of protein needed to fill all binding sites occurs at 20 nM protein, which gives a 1:1 binding stoichiometry.

#### IHF Junction Binding Affinity Is Not Altered by the Consensus Sequence

**Figure 2:**
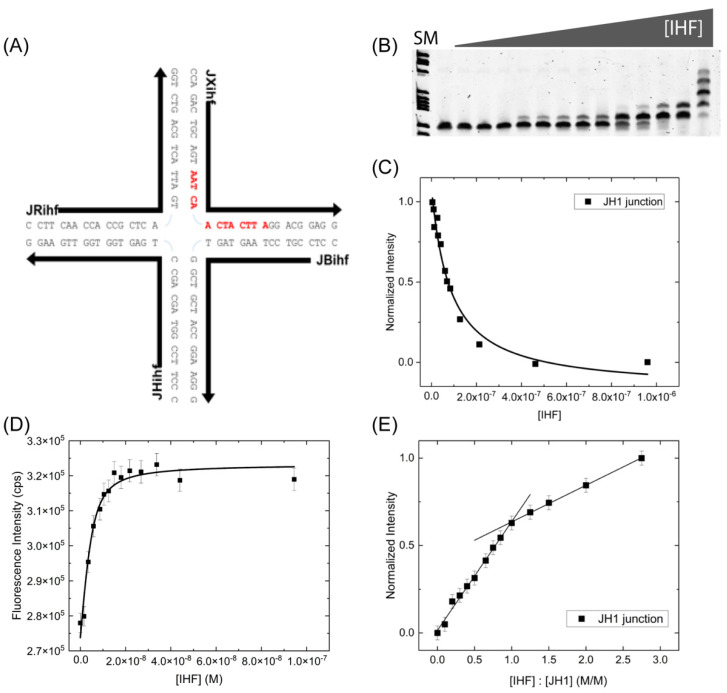
IHF binding to the JH1 junction. (**A**) Schematic of the JH1 junction with the IHF consensus binding sequence shown in red. (**B**) EMSA of the IHF binding to the JH1 junction at a constant concentration of 10 nM. SM indicates size marker, followed by free DNA lane, IHF is titrated in from 0–960 nM at the following concentrations: 4.5, 10, 12.5, 26.5, 30, 41.5, 60.5, 70, 83.5, 127, 212, 462, 960 nM (**C**) Analysis of the free DNA band with a 1:1 binding model yields an apparent *K*_D_ of 65 ± 8 nM. (**D**) Fluorescence intensity binding experiment measured with an end labeled JH1 junction yields a *K*_D_ of 9.2 ± 2.0 nM. (**E**) Stoichiometry binding experiments performed with a JH1 concentration of 100 nM yields a 1:1 binding ratio. All binding measurements were performed in a 70 mM KCl, 0.1 mM EDTA, 0.5% ethylene glycol, 5 mM Tris-HCl, pH 7.5 buffer. Error bars represent the SD from at least 3 experiments.

We explored the effect of the consensus binding sequence on IHF junction affinity by incorporating the H1 consensus sequence into the J34 junction [39] (Figure 2A). The 13 bp consensus sequence, which has the general form of WATCARNNNNTTR (W is A or T, R is A or G, N is any nucleotide), is incorporated into two arms of junction across the junction central region within the sequence context of the immobile J34 junction. As shown in Figure 2B, multiple bands are observed when measuring the affinity by EMSA and bound species appear early on at low protein concentrations, yielding a *K*_D(app)_ of 65 ± 8 nM (Figure 2C). This *K*_D(app)_ value is well within the range of that observed with the J34 junction and suggests that the incorporation of the consensus sequence does not significantly alter IHF binding affinity for the junction.

We have further investigated the binding affinity and stoichiometry using fluorescence intensity measurements (Figure 2D,E). Relative to the J34 junction, IHF exhibits a slightly lower binding affinity for the JH1 junction in solution with a measured *K*_D_ value of 9.2 ± 2.0 nM, which is within error of the J34 *K*_D_ values. As shown in Figure 2E, IHF binding stoichiometry to the JH1 junction is also 1:1, as for J34. Interestingly the slope of the plateau region is suggestive of a greater degree of non-specific binding. We speculate that this binding mainly occurs to the arms of the junctions, which resemble B-form DNA particularly when co-axially stacked [20,21,43,44]. Collectively, these data demonstrate the IHF binding affinity for junctions is not strongly influenced by the inclusion of the consensus sequence. As noted in other studies, IHF recognition and function does not always rely on the presence of the consensus sequence [45,46] and we suggest that in the case of IHF binding to junction the consensus sequence similarly does not play a large role, suggesting a structure-based recognition mechanism.

### 2.2. IHF Binding Alters the Conformation of the Junction

**Figure 3:**
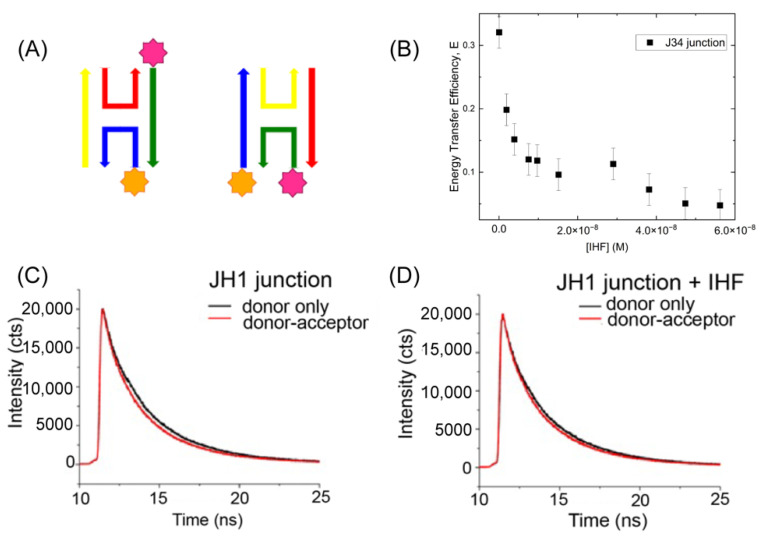
Förster Resonance Energy Transfer Measurements examining junction conformation. (**A**) Schematic of the labeling scheme employed illustrating that energy transfer is observed for the iso II conformation (X-strand donor, R-strand acceptor) (**B**) Energy transfer efficiency of the J34 junction as a function of IHF concentration using 20 nM junction. (**C**) Time-resolved fluorescence decays of the JH1 junction labeled with either the donor dye only or the donor-acceptor pair measured with a 300 nM concentration of JH1. (**D**) As shown in (**C**) but with IHF bound. Analysis of these decays reveals that IHF opens the junction. All measurements were performed in the presence of 70 mM KCl, 5 mM Tris, pH 7.6, 0.5% ethylene glycol. Error bars represent the SD from at least 3 different experiments.

Given the importance of junction conformation with respect to function, we further elected to investigate the structure of the junctions upon protein binding. We performed IHF measurements in the presence of 60–70 mM KCl, previously identified as relatively stringent binding condition [47]. At this salt concentration the junction adopts structures where the arms are co-axially stacked in two possible conformational isomers, referred to as iso I and iso II (Figure 3A) [15,28,29]. As previously shown by Lilley and co-workers, the J3 junction exists in an 80:20 iso II: iso I conformer distribution [23,28,29]. Thus, we covalently attached fluorescent labels to the R and X arms of the junction through a C6 linker to monitor junction conformation. This placement of the labels is expected to lead to high energy transfer efficiencies when the junction is in the iso II conformation, which is the expected dominant conformation in the absence of protein Figure 3A). Using fluorescein (FAM) as the donor dye and rhodamine (TAMRA) as the acceptor dye, we obtained a transfer efficiency of 0.35 corresponding to a distance of approximately 60 Å between the two arms as predicted by the iso II conformation (Figure 3B). Addition of increasing concentrations of IHF decreases the energy transfer efficiency, suggesting that IHF binding redistributes the population from the iso II conformation to either the open or iso I conformation (vide infra Section 2.3 and Section 2.4). In our labeling scheme with an R_0_ or Förster distance of 50 Å, we cannot readily distinguish between the open and iso I conformations due to the low transfer efficiencies in either orientation.

#### IHF Binding Alters the Conformation of the JH1 Junction

We further investigated the conformation of the JH1 junction with IHF bound and found that as with the J34 junction, IHF binding alters junction conformation. As the JH1 junction was not well characterized in terms of conformer populations and orientation, we employed time-resolved (TR) fluorescence spectroscopy to determine the relative distribution of junction conformations with and without IHF (Figure 3C,D). The FAM-labeled JH1 junction exhibits three lifetimes in the fluorescence decay: a relatively fast lifetime (0.2–0.6 ns), a mid-range lifetime (1.0–2.0 ns) and a slow lifetime (4–5 ns). These lifetimes are distinct and well-resolved by our system which has an instrument response function of 275 ps. The complexity in the lifetimes relative to the monomer arises due to the attachment of the dye to the ssDNA. By deconvolving the fluorescence decays into the respective lifetime components, we elucidated the relative populations of junction conformers and the effect of IHF on junction conformation (Supporting Material: Figure S1 and Table S1). The labeling scheme that we employed (Figure 3A) preferentially investigates the iso II conformation of the junction, as this is the orientation in which the labeled arms (R and X) are closest together in space. Given our labeling scheme, we assign the two faster lifetime components to the iso II and possibly open conformation (see below) and the iso I conformation is associated with the longest lifetime as the ends of the labeled arms are farthest apart in this configuration (Table 1). These assignments lead to a relative population distribution of 0.69 for the iso II conformation and 0.31 for the iso I conformation (Supporting Material: Table S2). This distribution is different from that observed for the J3 junction [29,31], which is not surprising given the different sequence at the junction center, which has been shown to be significant for determining conformer distribution [48,49].

We found that the population of the mid-range component increases from 0.23 to 0.32 upon IHF binding, which is tempting to attribute to an increase in population of the open conformation. An assignment of the open conformation to the mid-range component is not appropriate; however, as an examination of the lifetimes revealed that they all decrease in the presence of acceptor, thus each lifetime cannot be unequivocally assigned to one conformation. We instead examined the energy transfer in the system using the intensity-weighted lifetime, which weights each lifetime by the relative amplitude. In this analysis, we found that in the absence of protein, for the donor only and donor-acceptor samples, the mean lifetime changes from 1.81 to 1.43 ns, while with IHF bound, the intensity-weighted lifetime changes only slightly from 1.79 to 1.72 ns. These changes correspond to an efficiency of 0.21 without protein and less than 0.05 with protein (Table 1). The relatively significant decrease in FRET efficiency upon protein binding is attributed to the junction arms moving from the stacked iso II conformation to an open or low FRET orientation. The lower FRET efficiency is consistent with IHF stabilization of either the open or the iso I conformation.

### 2.3. IHF Binding Affinity Is Independent of Junction Arm Length: The J20 Model Junction

**Figure 4:**
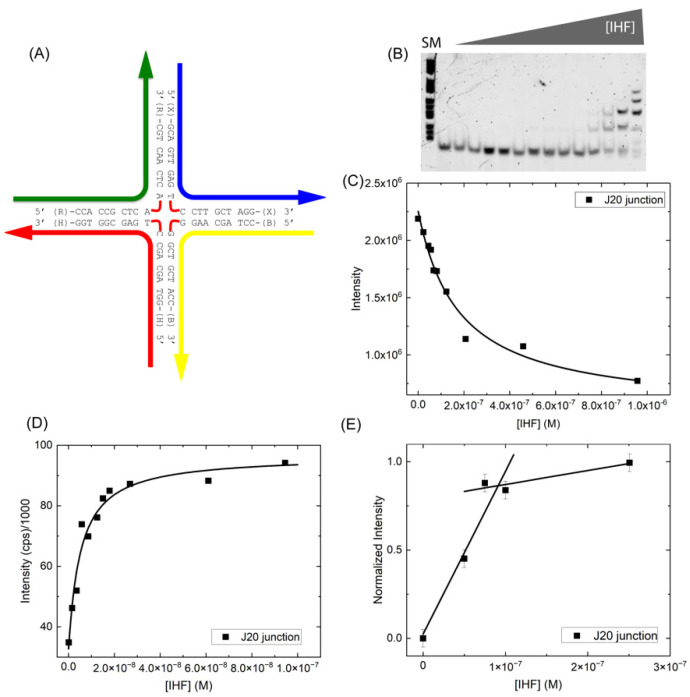
IHF Binding to the J20 junction. (**A**) Schematic of the J20 junction with sequence shown. (**B**) EMSA of IHF binding to the J20 junction at a constant concentration of 5 nM. SM indicates the size marker, the next lane is free DNA and the protein is titrated from 0–958 nM at the following concentrations: 24, 45, 56, 68, 82, 123, 208, 459, 958 nM. (**C**) Analysis of the free DNA band with a 1:1 binding model yields a *K*_D(app)_ of 170 ± 40 nM. (**D**) Fluorescence intensity measurements with a TAMRA 5′-end labeled junction yields a *K*_D_ of 4.4 nM. (**E**) Stoichiometry experiments performed with 100 nM of TAMRA end-labeled junction and increasing concentrations of IHF support a 1:1 binding model. All measurements were performed in the IHF binding buffer.

To improve detection of the open conformation in our measurements and address the nature of the IHF-induced conformational changes, we generated a junction with four 10 bp arms or 20 bp strands (J20) (Figure 4A). This junction based on the J34 junction construct (Figure 1B) potentially allows for experimental observation of the junction open conformation and can be used to explicitly explore how junction arm length affects binding affinity. As shown in Figure 4B,C, when measured in the gel, we observed a considerably lower binding affinity of IHF to the J20 junction, with a *K*_D(app)_ of 170 ± 40 nM. In appearance however the gel is quite similar to that observed with the J34 junction, with multiple bands observed at higher protein concentrations. In solution measurements using fluorescence intensity, we measured a significantly tighter binding affinity (*K*_D_ = 4.4 ± 0.5 nM), suggesting that IHF binds with high affinity to this junction with 20 bp arms (Figure 4D). This almost 50-fold difference in binding affinity between the gel and solution measurement potentially results from dilution effects in the gel; often binding affinities as determined by EMSA can be lower than those determined by fluorescence intensity or anisotropy [51,52,53,54]. The binding affinity in the gel may also be affected by the conformational dynamics of the junction as discussed below.

We note that when measured in solution, IHF affinity for the J20 junction (Figure 4) is comparable to that for the J34 junction, which has 17 bp arms, indicating that junction arm length is not a significant factor in determining IHF binding affinity for HJ substrates. Junction arm length probably determines the amount of non-specific binding, which is reflected in the slope of the plateau region in the stoichiometry plots. We also found that similar to the J34 junction, IHF binds to J20 with a 1:1 binding stoichiometry (Figure 4E). Given that IHF exhibits comparable binding affinity for both J34 and J20, we speculate that IHF binds to the central region of the junction and recognizes the junction in an architectural manner, that is due to the significant distortion of the junction arms from B-form DNA in the open form [4,5,55]. This recognition of bent DNA is consistent with IHF binding to duplex DNA, where it induces bends in sequences with or without the consensus sequence [45].

#### 2.3.1. Identification of IHF-Bound Junction Conformational States: J20 Junction

**Figure 5:**
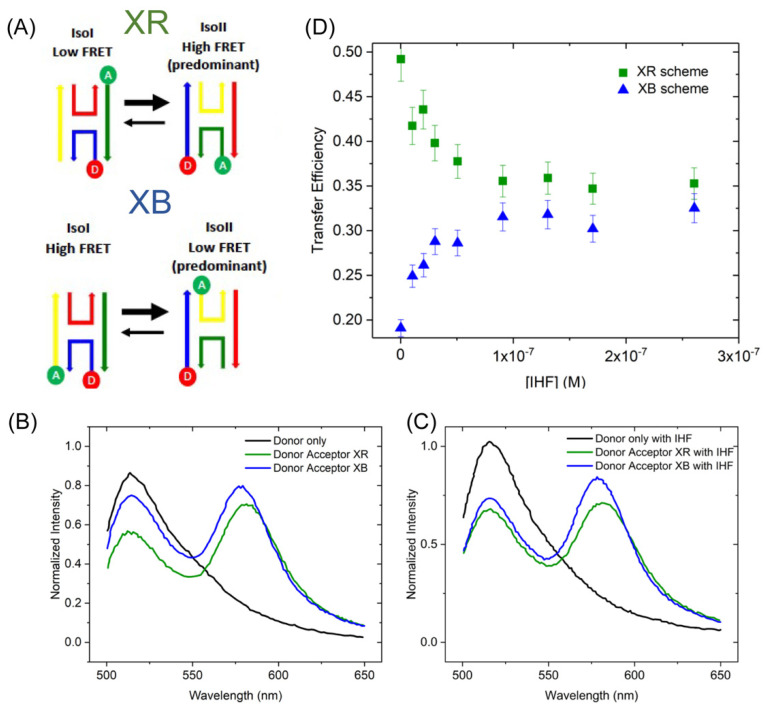
Steady State FRET analysis of IHF binding to the J20 junction. (**A**) Schematic of the two labeling schemes (XB and XR) employed to differentiate between the two possible conformations of the stacked junction. (**B**) FRET measurements of the junction alone, donor only shown in black, XR labeled junctions shown in green and XB labeled junctions shown in blue. (**C**) FRET measurements of the J20 junction in the presence of 275 nM of IHF. Traces are colored as in (**B**). (**D**) Energy transfer efficiency calculated as described in Materials and Methods as a function of IHF concentration for the XR (green) and XB (blue) labeling schemes.

To refine our assignment of the three different junction conformations by FRET experiments, we employed two different labeling schemes (Figure 5A). The labeling scheme in which the JX arm is labeled with fluorescein and the JB arm is labeled with rhodamine (JX-FAM, JB-TAMRA hereafter referred to as XB) yields higher transfer efficiencies for the iso I configuration than either the open or iso II. Conversely, the labeling scheme in which the JX arm is labeled with fluorescein and the JR arm is labeled with rhodamine (JX-FAM, JR-TAMRA referred to as XR) preferentially monitors the iso II conformation (Figure 5A). The greater energy transfer of the XR scheme can be seen in the relative ratios of the donor and acceptor peaks, where the acceptor peak at 560 nm is considerably higher than the donor peak in the absence of IHF. As shown in Figure 5B, for the XB scheme, the peaks are nearly equivalent in intensity. With the addition of protein, energy transfer decreases for the XR scheme as qualitatively observed by a loss of intensity in the acceptor peak; whereas, for the XB scheme energy transfer increases with a higher intensity of the acceptor peak and lower donor peak intensity (Figure 5C). Transfer efficiency is determined from the ratio of the donor fluorescence in the presence and absence of acceptor as described in Section 4.

Figure 5D depicts how the FRET efficiency, a proxy for junction conformation, changes with increasing concentrations of IHF. The relative distribution of the iso I and iso II conformations of the J20 junction were determined using time-resolved fluorescence spectroscopy as described below (Section 2.3.2). In those analyses, we found that the J20 junction has a similar distribution of conformers as the J3 junction, that is the iso II conformation is favored and 80% of the junctions are in that conformation (Table 2). For the steady state FRET measurements, the population distribution of the junction, which favors the iso II conformation, determines the efficiency outcomes for the different labeling schemes. In the absence of protein, labeling of the X and R strands leads to high transfer efficiencies (~0.5), as this scheme preferentially reports on the iso II conformation. In contrast, the XB labeling scheme leads to relatively low transfer efficiencies (0.2), as the population in the iso I conformation is relatively low (20%). This can be qualitatively observed in the emission spectra, IHF addition leads to either a decrease in energy transfer for the XR labeling scheme or an increase in energy transfer for the XB labeling scheme. As shown in Figure 5D, the relative changes in energy transfer result in comparable energy transfer efficiencies of ~0.35 for the final protein bound states of either labeling scheme. This relative increase (XB scheme) or decrease (XR scheme) in energy transfer is consistent with IHF binding resulting in a higher population of the open conformation relative to the other possible conformations.

#### 2.3.2. IHF-J20 Conformational States: Time-Resolved Förster Energy Transfer

To further explore the conformational populations of IHF-bound junctions, we employed time-resolved FRET to analyze the junction conformations. As with the steady state measurements, we monitored the conformations with both labeling schemes, XB and XR, and obtained three decay components similar to the JH1 junction. As shown in Table 2, the presence of protein does not significantly alter the donor only kinetics and the largest contributors to the fluorescence quantum yield are the long and mid-range lifetime components. Since the shortest lifetime component contributes the least to the quantum yield, it is the hardest to resolve from the other components and is possibly not completely resolved from the medium range component in our analysis. As we observe all three components in the donor only sample, the different components are not a consequence of energy transfer and the presence of the acceptor.

In the absence of protein, the decay kinetics are largely consistent with known population distributions of the conformers. Specifically, in the case of the XR labeling scheme without IHF, we attribute the slowest component to the iso I conformation with a normalized amplitude of 0.21. This is consistent with the previous results from Ha, Lilley and co-workers who observed an 80:20 ratio of the iso II to the iso I conformation for the J3 junction which is the template for the J20 junction [28,29,31]. The combination of the fast and the mid-range amplitudes yields the population attributed to the iso II conformation. In the XB labeling scheme, the fastest component is expected to correspond to the iso I conformation. The relative amplitude for this component is 0.26, which is close to the expected value of 0.20 [28,29,31]. We note that in contrast to the XR labeling scheme, in the XB scheme the smallest population is associated with the shortest lifetime, which makes it difficult to detect as it represents less than 10% of the total fluorescence quantum yield.

Despite this limitation, for both labeling schemes, we find that the trends are consistent with those observed using steady state fluorescence spectroscopy, that is an efficiency decrease for the XR scheme and an efficiency increase for the XB scheme, as suggested by the ratio of the amplitude-weighted lifetimes (Table 2). As more than one lifetime changes when we include the acceptor or add protein, we employ the amplitude- weighted lifetime to describe the energy transfer [50]. In the absence of IHF, the XR scheme yields the shortest <τ> or highest efficiency, consistent with 80% of the population in the iso II configuration or the high energy transfer form. With protein bound, the amplitude or relative population of the mid-range component increases, and energy transfer is reduced. With the XB scheme without IHF, the iso II conformation is the low FRET state, thus the transfer efficiency of the total population is relatively low. Addition of protein leads to an increase in transfer efficiency (0.14 to 0.29), which aligns with more junctions adopting either the open or the stacked iso I configuration. Importantly, the relative transfer efficiencies obtained in the presence of IHF for both labeling schemes are approximately the same (0.23 and 0.29), similar to what we observed using steady state fluorescence. The time-resolved fluorescence results support our finding that IHF addition alters junction conformation. Investigation of this conformational change using steady state and time-resolved fluorescence spectroscopic methods strongly points to a model in which IHF binding changes the junction population from the stacked configuration to an open one, resulting in reduced transfer efficiencies for the XR scheme and increased transfer efficiencies for the XB scheme (see Table 2 and Figure 5).

Although we do see an increase in the population of the mid-range component, the decays remain heterogeneous; therefore, we cannot unequivocally assert that only one conformation is present. Given our inability to assign one component of the decays to the open conformation and the fact that we observe changes in multiple decay or lifetime components upon protein binding, we chose to use single molecule fluorescence spectroscopy to fully resolve the different junction species present with IHF bound. Significantly, single molecule experiments remove any concerns regarding donor only species interfering with the energy transfer measurements, as only those molecules labeled with acceptor are monitored.

### 2.4. Single Molecule Fluorescence Experiments Identify Three Junction States and Confirm That IHF Binds to Open Junctions

**Figure 6:**
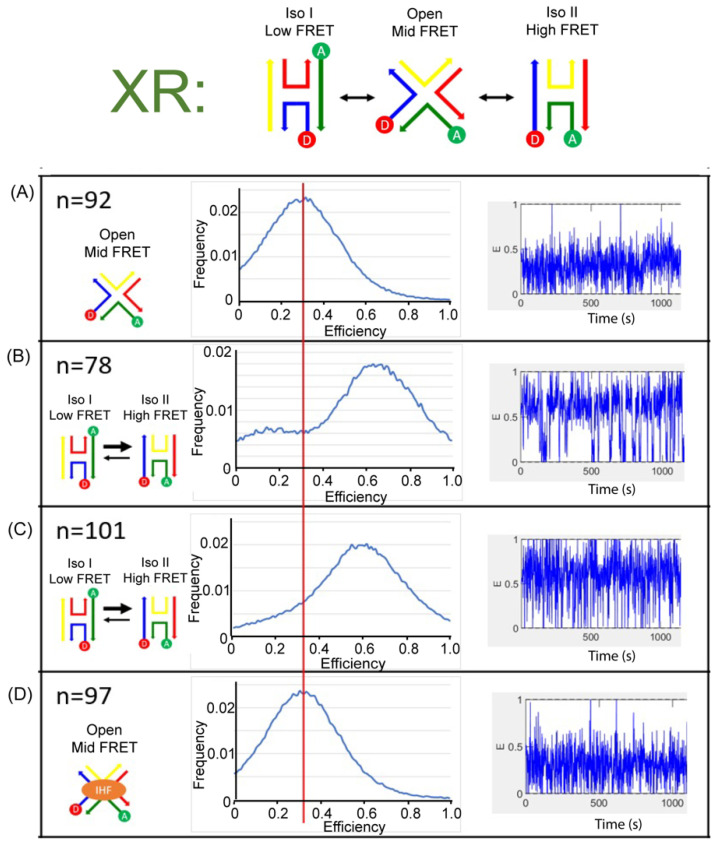
Single molecule fluorescence histograms (middle) and representative FRET time trajectories (right) of the J20 junction labeled with the XR scheme (left). (**A**) J20 junction in the presence of 1 mM EDTA. (**B**) J20 junction in the presence of 50 mM Mg^2+^ (**C**) J20 junction in the presence of 1 mM Mg^2+^ (**D**) J20 junction with IHF bound in 1 mM Mg^2+^. All measurements were done in the imaging buffer as described in the Materials and methods. N-value shows the number of individual molecule trajectories used to construct the population histograms.

**Figure 7:**
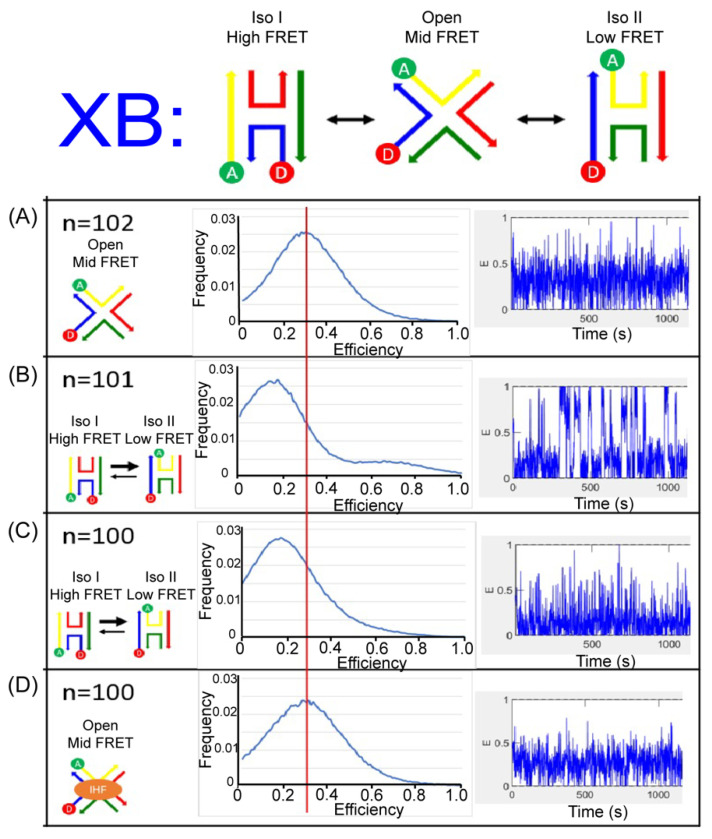
Single molecule fluorescence histograms (middle) and representative FRET time trajectories (right) of the J20 junction labeled with the XB scheme (left). (**A**) J20 junction in the presence of 1 mM EDTA. (**B**) J20 junction in the presence of 50 mM Mg^2+^ (**C**) J20 junction in the presence of 1 mM Mg^2+^ (**D**) J20 junction with IHF bound in 1 mM Mg^2+^. All measurements were done in the imaging buffer as noted in the Materials and Methods. N-value shows the number of individual molecule trajectories used to construct the population histograms.

We monitored the J20 junction using single molecule FRET measurements to distinctly identify the three states of the junction and the conformation induced with IHF binding. We used a biotin-streptavidin linkage to immobilize labeled junctions on a functionalized glass surface within a home-built microfluidic device, and used two-channel total-internal reflection fluorescence (TIRF) microscopy to simultaneously capture fluorescence intensity traces for individual pairs of donor and acceptor dyes. For these experiments, we employed the Cy3 and Cy5 dyes as the donor and acceptor labels for better photostability. As shown in Figure 6 and Figure 7, we employed the same labeling schemes (XR and XB) to monitor the conformational dynamics of the junction. As shown in the representative time-based trajectories, junction transitions are fast and frequent under certain conditions. We investigated junction dynamics at 1 mM and 50 mM Mg^2+^, in the absence of Mg^2+^ (1 mM EDTA) and in the presence of bound IHF (Supplementary Materials: Figure S2). In the presence of EDTA, the FRET efficiency time traces obtained from single junctions fluctuate rapidly for both the XR and XB labeling schemes, but these fluctuations appear to be centered around an intermediate FRET efficiency value (Figure 6A and Figure 7A). We made histograms of all observed efficiencies for these single-molecule FRET trajectories, which reveal that the junctions primarily exhibit an intermediate FRET efficiency of 0.3, with very few observations of FRET efficiencies above 0.6. For both labeling schemes, individual junctions mostly exhibit rapid fluctuations around this intermediate FRET state, although excursions into a high FRET state occasionally occur.

In the presence of 50 mM Mg^2+^, we detected two peaks in the histogram of observed FRET efficiencies. For the XR labeling scheme, the high FRET state has the higher peak, centered at an efficiency of about 0.7. For the XB labeling scheme, the low FRET state is dominant, with the peak centered at an efficiency of about 0.2. Neither labeling scheme results in a peak in the FRET efficiency histogram matching the intermediate FRET state observed in the absence of Mg^2+^. Thus, for the XR labeling scheme we assign the high FRET state to iso II and the low FRET state to iso I. For the XB labeling scheme, we assign the high FRET state to iso I and the low FRET state to iso II. In both labeling schemes, the state observed in the presence of EDTA is considered the open state, with an intermediate FRET value of 0.3. In the FRET efficiency time traces, the dynamics are considerably slower in the presence of 50 mM Mg^2+^ and transitions between high and low FRET states can be identified (Supplementary Materials: Figure S3). For the XR labeling scheme, individual junctions spend more time in the high FRET state (Figure 6B), while for the XB labeling scheme junctions spend more time in the low FRET state (Figure 7B). This indicates that, although junctions are able to transition between different stacked states under these conditions, individual junctions spend more time in the iso II conformation.

The FRET time trajectories reveal that at the lower concentrations of Mg^2+^ used, such as 1 mM, the transitions between the conformers become significantly faster (Figure 6C and Figure 7C). Once the concentration of Mg^2+^ was reduced below 10 mM, we could no longer resolve transitions between high and low FRET states (Supplementary Materials: Figure S3). The fast time scale of the transitions, which is on the order of the instrument time resolution, leads to broader peaks and reduced resolution of individual states in the FRET histograms. This transition behavior with the associated dependence on Mg^2+^ concentration is consistent with earlier reports of junction dynamics [29,56,57] and indicates that the J20 junction behaves similarly to the J3 junction.

As shown in Figure 6D for the XR scheme, addition of the IHF protein in the presence of 1 mM Mg^2+^ leads to the adoption of an intermediate state, comparable to that observed in the presence of EDTA. A similar effect is observed for the XB scheme, where the population distribution also yields an intermediate FRET value with IHF bound (Figure 7D). The similarities in FRET values under the two labeling conditions argues that IHF binding either stabilizes or induces the open state. Although the median FRET value is at an intermediate point between the two stacked conformations, the time traces reveal that the junction arms fluctuate considerably with IHF bound and the junction occasionally transitions to the iso I or the iso II conformation.

Under conditions where the transitions are very rapid (e.g., 1 mM Mg^2+^) it was difficult to discern the transition rates between the two conformers; therefore, we applied the methodology of cross-correlation analysis [58,59], to determine the overall transition rates (Supplementary Materials: Figure S4. Given the relatively fast rates observed, individual steps in the conformer transition were not resolved and the rate reported is the overall transition rate including any intermediate steps. As expected for donor and acceptor dyes that are fluctuating, but constrained to remain in proximity to each other, all traces in which both donor and acceptor were present displayed a negative cross-correlation between donor and acceptor intensities. The analysis of J20 in 1 mM EDTA did not show any change in cross-correlation value as the time shift increased, implying that the junctions were in a static open form, or that any fluctuations were too rapid to be resolved. For J20 in the presence of Mg^2+^, the analysis showed that the cross-correlation values decreased in magnitude as the time shift increased, indicating that the junctions were transitioning between high and low FRET states (Supplementary Materials: Figure S4). In the presence of both IHF and Mg^2+^, a similar pattern was observed, with a relaxation to a lower magnitude of cross-correlation as the time shift increased. This indicates that the IHF-bound junction remains dynamic. Cross-correlation analysis indicates that in contrast to the junction prepared with 1 mM EDTA, the IHF-bound Holliday Junctions do undergo transitions between at least two states, even though the FRET histograms closely resemble the static open form.

**Figure 8:**
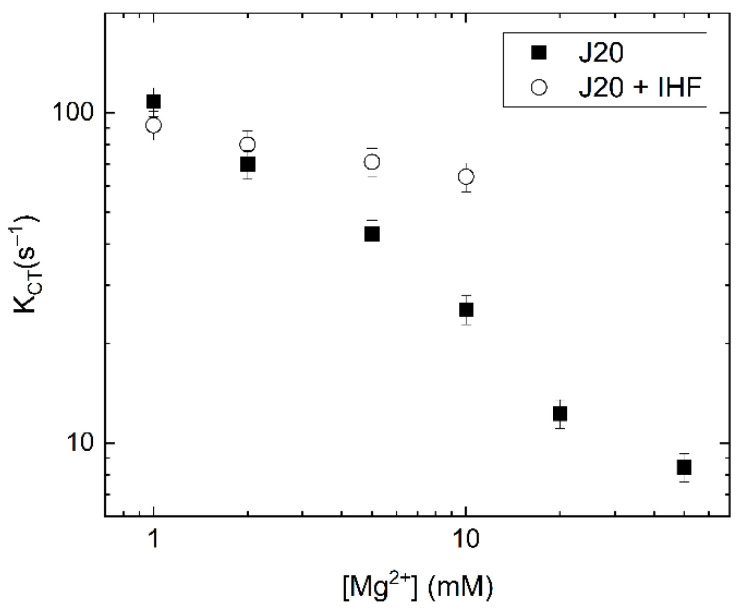
Conformer transition rates determined from cross correlation analysis of single molecule traces as a function of Mg^2+^. Sample traces shown in Figure 6 and Figure 7. The J20 junction (solid squares) exhibits a steep dependence on Mg^2+^ concentration with slower rates occurring at higher concentrations. The dependence becomes shallower in the presence of IHF (open circles), consistent with IHF-dependent stabilization of the open form.

We fitted the cross-correlation relaxations with a single exponential, yielding a time constant that reports on the conformer transition rate. This conformer transition rate was sensitive to the concentration of Mg^2+^ (Figure 8). For free junction, conformer transition rates decreased by an order of magnitude as the concentration of Mg^2+^ was increased from 1 mM to 50 mM. This trend is consistent with the stabilizing effects of Mg^2+^ on the stacked forms of the junction with relatively long ion residence times in the junction central region [21,24,56,57]. Similar to free junction, the conformer transition rates for the IHF-bound junction also decrease with increasing Mg^2+^ concentrations. However, the dependence is much less pronounced, with only a slight decrease, from 90 s^−1^ to 70 s^−1^, as the concentration of Mg^2+^ increased from 1 mM to 10 mM. We were unable to confirm stable IHF binding at concentrations of Mg^2+^ higher than 10 mM.

At low concentrations of Mg^2+^, the conformer transition rates for free and IHF bound junctions were similar, while at higher Mg^2+^ concentrations, the transition rate with IHF bound was faster than what was observed for the free junction. These findings indicate that IHF binding modulates the conformer transition rate, facilitating rapid fluctuations under conditions that would normally result in decreased dynamics. This is consistent with our hypothesis that IHF stabilizes the open junction form, which acts as an intermediate during the iso I to iso II transition. We note that, as is the case for free junctions in the presence of lower Mg^2+^, the observed transitions with IHF bound are relatively fast, and we are not able to identify individual transitions in the FRET efficiency trajectories. Therefore, we cannot say with any certainty how many states IHF-bound junctions visit, though the dominant state appears to be the open state. Although the significance of the open form as an intermediate in conformer transitions needs to be verified with better time resolution, the effect of bound IHF on the junction transition rates does suggest that the junction visits the open form during the conformer transitions.

### 2.5. Dynamics of the IHF-J20 Binding Interaction

**Figure 9:**
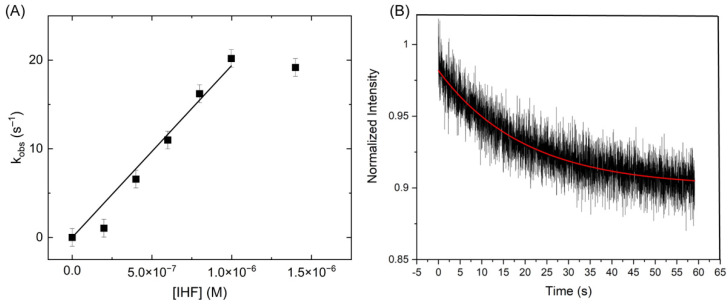
Kinetic analysis of IHF binding to the J20 junction. (**A**): IHF binding is measured through FRET, yielding observed binding rates, which increase until the binding is saturated at 1 μM IHF. These rates measured as a function of IHF concentration yield an on rate of 1.93 × 10^7^ M^−1^s^−1^. (**B**): An excess amount of unlabeled junction was introduced to measure an off rate of 0.05 s^−1^.

Given the dynamic nature of the junction under protein binding conditions, we sought to address whether the protein remains bound during the conformational transitions. To examine IHF binding kinetics to the junction, we performed stopped flow experiments using the FRET signal (donor increase or acceptor decrease) to monitor IHF binding. Although the binding interaction is bimolecular, these measurements were performed with an excess of IHF, in order to treat the reaction as pseudo first order [60]. Thus, by measuring the observed binding rate as a function of IHF concentration, we were able to determine the bimolecular on rate for the reaction. As shown in Figure 9, we found that as the IHF concentration increases, the on rate also increases until it plateaus at approximately 1 μM IHF with a rate of approximately 20 s^−1^. This overall behavior is similar to that observed with the consensus duplex as measured previously [35] and by us (Supplementary Materials: Figure S5).

Analysis of these observed rates yields an on rate of 1.93 × 10^7^ M^−1^s^−1^ for the junction complex, which is an order of magnitude slower than that observed for the consensus duplex. The observation of a plateau at high protein concentration suggests that under these conditions the rate of binding becomes equivalent to a second rate, most likely associated with a conformational change. In previous studies with IHF bound to the H’ consensus duplex, the same behavior was observed and this rate was attributed to DNA bending [35]. Using temperature jump and stopped flow methods, Ansari and co-workers determined that IHF exhibits biphasic kinetics when binding to a consensus duplex and they attributed the slow phase in binding to formation of a specific complex between IHF and the duplex [36,61]. The initial diffusion-limited encounter with the DNA was attributed to a non-specific binding step. Thus, we attribute the slower rate observed in the IHF-junction binding kinetics to a conformational change of the junction to the open state and invoke a similar mechanism of binding and recognition. In this model, formation of the specific IHF-open junction complex is slow relative to the initial binding interaction, which is non-specific in nature. Single molecule IHF-J20 measurements yield a conformer transition rate (k_ct_) of 80–90 s^−1^ (Figure 8), which is approximately 4× faster than the limiting k_obs_ rate in the stopped flow experiments under the same conditions (Figure 9). This similarity in observed association rates is consistent with our model where the limiting on rate for the IHF-junction complex reflects in part the time needed for the junction to adopt the open state.

Dissociation kinetics can reveal further information about the IHF-J20 complex where a fast protein off rate (k_off_) would imply that the observed junction conformational transitions are occurring without IHF bound. We measured the protein off rates of the IHF-J20 complex using a competition assay [60]. In this case, we introduced an excess amount of unlabeled junction to an equilibrated solution of IHF bound to labeled junction and monitored the loss of signal (Figure 9B). The decays were well described by a single exponential and yielded an off rate of 0.05 s^−1^, 100-fold slower than the slowest junction transition rate measured of approximately 5 s^−1^, and well over 1000-fold slower than the conformer transition rates observed in the presence of IHF (Figure 8). This junction off rate (0.05 s^−1^) is comparable to that previously observed for the consensus duplex (0.01–0.07 s^−1^) [35]. Thus, from a comparison of the k_off_ and k_ct_ rates it can be inferred that IHF remains bound to the junction during the conformational transitions and further suggests it does not lock the junction into the open conformation when bound. These results also indicate that the protein remains bound while the junction continues to access different conformations and suggests that the protein may form a partially dissociated state, as recently observed with RuvC and other proteins [37,62]. Although the single molecule time traces would suggest that full conversions from one conformation to the other do not occur frequently, the cross-correlation analysis suggest that conversions between the open state and at least one of the stacked isoforms does occur. Importantly, we recognize that the shorter arm lengths used in our J20 model junction, which help us to identify the different conformational states, may be more dynamic than junctions in vivo with considerably longer arms.

## 3. Discussion

### 3.1. IHF Binds to and Stabilizes Open Junctions–A Mechanism for Facilitating Recombination?

In this study we have examined the interaction of the IHF protein with three different junctions. For all junctions, we observed nanomolar binding affinity and our different constructs all form a 1:1 IHF:junction complex. Variations in our construct demonstrate that the length of the arm or the introduction of the consensus sequence does not alter the binding interaction, strongly indicating that the protein binds to the center of the junction. FRET measurements consistently point to IHF binding to and stabilizing the open conformation of the junction. This form of the junction is capable of branch migration and is the junction conformation recognized by many resolvases prior to cleavage [25,63]. Although the functional role of the IHF-junction complex is not fully known, IHF stabilization of the open conformation may be significant for recombination events. In *Pseudomonos putida* IHF facilitates homologous recombination and the occurrence of point mutations [32]. These types of genetic outcomes could be associated with IHF-dependent stabilization of the open junction conformation during recombination. IHF participates in many different functions in the cell; notably in addition to transcription and replication, IHF is implicated in site-specific recombination and transposition reactions which could be strongly influenced by the presence of stabilized open junctions. Additionally, biofilms in a variety of bacteria contain IHF and the protein has been shown to stabilize the eDNA [11,12]. IHF stabilization of the junction open conformation could be an important aspect of forming the DNA lattice needed for biofilm formation.

### 3.2. Dynamic Behavior of IHF-J20 Complex Potentially Signifies Formation of a Partially Dissociated State

Interestingly, smFRET time traces show that the junction arms while in the open form with IHF bound are quite dynamic. This dynamic behavior is suggestive of a partially dissociative state for the complex, in which the junction can still transition between conformers with IHF bound. Such partially dissociated states have been observed previously with DNA junctions by smFRET for the junction binding resolvase, T7 endonuclease I and RuvC [37,62]. In the case of T7 endonuclease case branch migration and conformer transitions were observed even in the presence of bound enzyme [62]. In our system, the junctions are non-migrating therefore only conformer dynamics are monitored by our FRET labeling schemes. The IHF off rate for the junction as measured by stopped flow confirms the presence of protein during the conformer transitions. Moreover, IHF stabilization of the open state facilitates these transitions, as shown by the differential change in rates as a function of [Mg^2+^] with and without IHF (Figure 8). The conformational flexibility observed upon IHF binding could potentially facilitate the binding of other proteins to complete other functions.

In contrast, IHF structural homolog HU binds to four-way junctions in a 2:1 ratio and stabilizes the stacked conformation [15]. These two nucleoid-associated proteins exist in high concentrations in the *E. coli* cell [64] and stabilize junctions in the open and stacked conformations, respectively. This difference in binding behavior is supported by the observation that in biofilms HU and IHF bind to different locations [11]. We speculate that regulation of junction conformation and function could be achieved through competitive binding between these two proteins.

### 3.3. Slow Association Kinetics Suggest IHF Captures Junctions in the Open Conformation

In this study we have shown that IHF binds to junctions with high affinity, and that IHF-bound junctions are biased toward the open conformation. One question is whether IHF induces the open conformation upon binding or captures the transiently populated open conformation. Although such questions are difficult to address experimentally, some of our data hints to a conformational capture mechanism, in which IHF binds to an open junction. Namely, the *K*_D(app)_ measured in the gel for the J20 junction is 50× lower than that measured in solution. Given our stopped flow data, the weaker affinity could be a result of slower conformational transitions in the gel and fewer opportunities to form complexes with an open junction prior to separation as a consequence of migration in the gel matrix. This effect becomes more pronounced in EMSA performed in the presence of 1 mM Mg^2+^ which significantly slows the conformer transitions relative to monovalent ions (Supplementary Material: Figure S2). The rate limiting step in the association reaction (Figure 9) appears to be a conformational one where the time constant is similar to what we observe for the junction conformational transitions under the same conditions (Figure 8). These observations are supportive of a model in which the initial encounter between IHF and the junction is non-specific, and the higher affinity complex is formed once the junction adopts the open conformation. This behavior aligns with current understanding of how IHF recognizes its consensus site in a DNA duplex and argues for a similar mechanism of binding and recognition.

## 4. Materials and Methods

Unless stated otherwise, all chemicals and materials were obtained from Sigma-Aldrich Chemical Company (St. Louis, MO, USA) or Millipore Sigma (Burlington, MA, USA).

### 4.1. IHF Growth and Purification

IHF was purified from *E. coli* strain 1084B containing an IHF overexpressing plasmid (generous gift from Stephen Goodman). A cell culture was started from a single colony grown overnight on an LB amp plate and was grown in LB medium containing 50 μg/mL ampicillin at 37 °C. The culture was induced with 0.04 mM isopropyl β-D-1-thiogalactopyranoside when the optical density OD_650_ reached 0.9. Following induction, the cells were grown until OD_650_ reached 2.6. The cells were harvested and resuspended in 20 mM Tris-HCl (pH 7.4), 20% sucrose solutions (*w*/*v*) and stored at −80 °C. All the subsequent steps were performed on ice at 4 °C. The cells were lysed in buffer A (20 mM Tris-HCl pH 7.4, 1 mM EDTA, 50 mM NaCl, 10% glycerol, 3 mM 2-mercaptoethanol (βME) with additional 1 M KCl and 1 nM phenylmethylsulfonylfluoride followed by three rounds of homogenization using an EmulsiFlex C5 homogenizer (Avestin, Ottawa, Canada). The cell lysate was centrifuged at 2988× *g* for 1 h. The clear supernatant was dialyzed overnight against buffer A. To further remove the nucleic acids bound to IHF, Polymin P precipitation of DNA was performed. Polymin P solution was gradually added to the dialyzed solution until a final concentration of 0.05% was achieved over a period of 20 min and the sample was stirred for an additional 20 min. The sample was centrifuged at 2988× *g* for 40 min. The pellet was resuspended with 35 mL of Buffer A with 500 mM NaCl to retrieve any remaining IHF associated with the precipitated DNA. The mixture was centrifuged at 2988× *g* for 20 min and supernatant was combined with the supernatant from the Polymin P precipitation.

Ammonium sulfate was gradually added to the supernatant to reach 0.242 g/mL over 20 min, and the solution was left stirring for an additional 30 min. The mixture was centrifuged at 2988× *g* for 40 min. Additional ammonium sulfate was added to the supernatant, reaching a final concentration of 0.564 g/mL over 20 min. The mixture was left stirring on ice for 90 min and centrifuged at 2988× *g* for 40 min. The pellet was resuspended in 20 mL of Buffer A and dialyzed against buffer A.

The dialyzed sample was loaded onto a HiTrap Heparin HP Column (GE Healthcare, Piscataway, NJ, USA). The column was equilibrated with buffer A, loaded with protein sample, washed with buffer A, and eluted with a linear gradient of 20 column volumes from 0.1 M to 1.7 M NaCl. IHF eluted at around 800 mM NaCl. The fractions containing IHF were combined and dialyzed against buffer A without βME and subsequently concentrated to 20 μM. The solution was centrifuged at 5856× *g* for 15 min to remove any aggregates. The concentration of the protein was determined by UV-Vis spectroscopy at 276 nm using the known extinction coefficient of 5800 cm^−1^M^−1^. The protein solution was mixed with an equal volume of glycerol and stored at −80 °C. Each stock aliquot was dialyzed against IHF binding buffer (5 mM Tris-HCl pH 7.4, 70 mM KCl, 0.1 mM EDTA, 10% ethylene glycol) prior to use.

### 4.2. Preparation of DNA Substrates

DNA single strands were obtained from Integrated DNA Technologies (Coralville, IA, USA). Obtained strands were either purchased HPLC-purified or gel purified by us as described [15]. Strands containing 6-MI were purchased HPLC-purified from Fidelity Systems (Gaithersburg, MD, USA). To prepare DNA duplexes, DNA strands were mixed in equimolar amounts in the annealing buffer (10 mM Tris-HCl pH 7.4, 0.1 mM EDTA pH 8.0 and 300 mM NaCl) and rapidly heated to 90 °C. After a 5 min incubation, the strands were allowed to cool in a water bath to room temperature over 12 hrs. To prepare the junctions, single strands were added in equimolar amounts in the annealing buffer and heated to 80 °C for an hour and half in the water bath. The water bath was then cooled down to 50 °C for four hours and then slowly cooled down to room temperature for 12 h. Proper formation and purity of DNA substrates was verified using nondenaturing gel electrophoresis. Samples that were greater than 90% were used for experiments.

### 4.3. Electrophoretic Mobility Shift Assay (EMSA)

EMSA was conducted as previously described [15,51] For gels containing Mg^2+^ a 6.5% native polyacrylamide gel (29:1) with 1 mM MgCl_2_ was used in a Tris-borate buffer (pH 8.3) also with 1 mM MgCl_2_. J20 and IHF were mixed in IHF Mg binding buffer (5 mM Tris-HCl pH 7.4, 1 mM MgCl_2_, 10% ethylene glycol). Visualization of DNA bands was done using SYBR Green1 (Thermo Fisher, Waltham, MA, USA) and a Typhoon Trio Variable Mode Imager (GE Healthcare Biosciences, Chicago, IL, USA). Image Quant (GE Healthcare Biosciences, Chicago, IL, USA) was used to determine the intensity of the free DNA bands. An apparent dissociation constant *K*_D_ was determined from the band intensity as a function of increasing protein concentration. Analysis was done as previously described, assuming a 1:1 protein/DNA ratio [15,51].

### 4.4. Fluorescence Intensity and Anisotropy

Fluorescence spectroscopy and anisotropy measurements were performed using a Horiba Spectromax-4 fluorometer (Edison, NJ, USA) as described previously [15]. Experiments were performed in IHF binding buffer at 10 °C. The sample volume was kept constant at 600 μL, adding more protein through titration while maintaining DNA concentration. Samples were excited at 490 nm and emission intensity was collected at 515 nm or 520 nm for single point data or scanned from 505 to 650 nm. Both excitation and emission used a 7 nm slit bandpass and integration time of 0.5 s. Measurements were repeated up to 10 times until data reached a standard error of 2% or below. Samples were incubated at 10 °C for 3 min with continuous stirring before acquisition of data.

Analysis of binding constants was performed assuming a 1:1 binding stoichiometry with the following equation:

(1) $$f_{b}=\frac{\left(P_{0}+D_{0}+K_{D}\right)-\sqrt{{\left(P_{0}+D_{0}+K_{D}\right)}^{2}-4\times P_{0}\times D_{0}}}{2D_{0}}$$

where *P* indicates protein, *D* indicates DNA and the subscript 0 indicates total concentration, *K_D_* is the dissociation constant, and *f_b_* is the fraction bound. We further define the fraction bound as:

(2) $$f_{b}=\frac{\left(i-i_{0}\right)}{\left(i_{f}-i_{0}\right)}$$

where *i* is the measured fluorescence intensity or anisotropy, *i*_0_ is the initial value and *i_f_* is the final value.

### 4.5. Steady-State Förster Resonance Energy Transfer

Steady-state FRET experiments were performed using 5′ end-labeled DNA substrates. Donor only junctions were labeled with 5-carboxyfluorescein succinimidylester (FAM) and donor-acceptor substrates were labeled with 5-carboxyfluorescein succinimidylester (FAM) and 5-carboxytetramethylaminorhodamine succinimidylester (TAMRA) (Invitrogen, Thermo-Fisher, USA). J34 and JH1 were measured in the standard IHF binding buffer.

All experiments with the J20 junction were performed in the IHF Mg^2+^ binding buffer at a concentration of 100 nM DNA. Protein was titrated into either the donor-only or donor-acceptor sample. The sample volume was kept constant at 600 μL. JH1 and J34 junctions were excited at 490 nm and the emission was monitored at 520 nm for single point measurements or scanned from 505 to 650 nm at a rate of 1 nm/pt with an integration time of 1 s. For J20, fluorescence emission spectra were obtained by exciting the donor dye (FAM) at 375 nm and scanning the emission at a rate of 3 nm/pt with an integration time of 1 s. Samples were contained in 5 × 5 mm glass cuvettes and maintained at 10 °C with constant stirring. Analysis of steady-state FRET was performed as previously described [15,51,65].

### 4.6. Time-Resolved Förster Resonance Energy Transfer

The time-resolved FRET data were acquired from the samples with the same labeling scheme and composition as described above in Section 4.4. The samples were incubated for 5 min at 10 °C and continuously stirred during the experiment. Donor-only data were acquired with the protein present to ensure that any observed quenching in FRET samples was caused by the presence of acceptor. The measurements were done using a time-correlated single photon counting instrument (TCSPC) (PTI TimeMaster instrument, Horiba, NJ, USA) with a Becker & Hickl 375 nm pulsed picosecond laser diode for sample excitation. Fluorescence decays were obtained at 515 nm with a 450 nm cutoff filter to avoid scattering excitation light. Data were collected in a 50 ns time window until counts in the peak channel reached 20,000. Excitation and emission slits were both set to a 15 nm bandpass. IRF data were acquired at the laser wavelength (375 nm) using a dilute scattering solution and an OD 2.0 neutral density filter. The intensity decay curves were fit to a sum of exponentials. The curves were analyzed and fitted with the FargoFit program created by Igor Negrashov [66] and Globals for Spectroscopy (https://www.lfd.uci.edu/globals/ (accessed on 16 November 2022)). Quality of the fits was evaluated through consideration of the chi-squared values (typically 0.8 < χ^2^ < 1.2) and visual assessment of the residuals.

### 4.7. Single-Molecule FRET with Total Internal Reflection (TIRF) Microscopy

Single-molecule experiments were performed in a microfluidic chamber, assembled by sandwiching SecureSeal adhesive sheet with a channel cut into it between a quartz slide with drilled inlet and outlet holes and a glass coverslip. The coverslip was functionalized with 3-aminopropyltriethoxysilane, then amine-reactive, high molecular weight PEG molecules were covalently attached to the surface. This PEG brush inhibits surface adsorption of biomolecules, and a small percentage of the PEG molecules were terminated with a biotin. Prior to building the microfluidic device, the functionalized surface was coated with streptavidin, to facilitate attachment of biotinylated DNA constructs. PE60 tubing was used for the inlet and outlet tubes, which were coupled to a syringe pump that controlled flow through the channel, as described previously [67]. The microfluidic chambers were incubated with a blocking buffer (20 mM pH 7.4 Tris-HCl, 100 mM NaCl, 1 mM EDTA and 0.4 mg/mL bovine serum albumin) for one hour after assembly, then placed on the inverted microscope for imaging. All data were acquired at room temperature in imaging buffer, which was comprised of 20 mM Tris-HCl pH 8.0, salts of indicated concentrations, 50 mM Trolox, oxygen scavenging system (165 U/mL glucose oxidase, 2170 U/mL catalase, 5 mg/mL glucose), 0.5% (vol/vol) ethylene glycol and 0.1 mg/mL BSA.

Biotinylated, Cy3-Cy5 labeled Holliday Junctions were flowed through the microfluidic channel at a concentration of 4 pM in blocking buffer, resulting in a sparse coating of junctions immobilized on the coverslips. The biotinylated JB arm of the HJ used in smFRET experiments had an additional six thymine bases added to the end to avoid constriction of junction movements by the coverslip surface. Excess junctions were washed away by flowing additional blocking buffer, the channel was prepared for the introduction of protein by flowing imaging buffer through it, then IHF protein was flowed through at a concentration of 100 nM in imaging buffer. After incubating the junctions with protein for 5 min, the unbound proteins were washed out with 3 chamber volumes of imaging buffer. The high concentration of protein ensured that the junctions were almost 100% bound, even after incubating in the imaging buffer for 2 h. The surface-immobilized junctions were illuminated with a 532 nm diode-pumped solid-state laser focused on the back-focal plane of the high numerical aperture 60× oil immersion objective to achieve through-objective TIRF imaging. The fluorescence signals of Cy3 and Cy5 were split by a 640 nm single-edge dichroic mirror and projected to two different regions of the Hamamatsu X2 EM-CCD camera chip. The donor and acceptor channels are aligned by imaging quantum dots with peak emission at 605 nm. The emission from these quantum dots can be seen in both imaging channels, so they can be used to determine the transform matrix needed to realign the two images. The time resolution was set at 13.9 ms, which provides the maximum attainable frame-rate for the camera.

The raw data was analyzed with the iSMS software package [68]. Apparent FRET efficiency (E_FRET_) was calculated from the fluorescence intensity of donor (I_D_) and acceptor (I_A_) using the formula: E_FRET_ = I_A_/(I_D_ + I_A_). Background noise and cross-talk were calculated as previously described [68,69]. Only molecules with both Cy3 and Cy5 signals were analyzed as FRET pairs, and pairs showing multiple bleaching steps were excluded from analysis. FRET efficiency histograms were generated directly from the calculated time traces. The calculated FRET efficiencies for every time point in the trace were included in the analysis, and bin widths were selected based on the number of data points available. Trajectory lengths generally permitted bin widths smaller than 0.01, for at least 100 bins covering the range of FRET efficiencies between 0 and 1.

Transition rate analysis was performed in the MATLAB platform. The transition rates of J20 in 50 mM and 10 mM Mg^2+^ were analyzed with dwell time analysis. For these data sets, the FRET efficiency traces were processed using the vbFRET algorithm [70] to identify state transitions. Subsequent exponential fitting of the dwell time distribution was done in OriginPro. Junctions observed in lower concentrations of Mg^2+^ or with IHF bound did not result in FRET trajectories with identifiable transitions, therefore all samples were also analyzed using cross-correlation analysis. The normalized cross-correlation between the time traces of the fluorescence intensity of the donor (*I_D_*) and acceptor (*I_A_*) fluorophores were calculated as a function of the time lag, Δ*t*, between data points:

(3) $$C_{cross}\left(\Delta t,\;t_{start}:t_{end}\right)=\sum_{t_{start}}^{t_{end}}I_{D}\left(t\right)I_{A}\left(t-\Delta t\right)/I_{D}\left(t\right)I_{A}\left(t\right)$$

The intensities of each trace were normalized before analysis and each cross-correlation analysis was conducted with the concatenated trajectories of more than 50 FRET pairs. The resulting cross-correlation curves were all fit to a single exponential function, where the decay constant was taken to be the relaxation time for the cross-correlation or the inverse of the overall conformer transition rate, which encompasses all transitions that occur within the timescale observed.

### 4.8. Stopped Flow Experiments

Stopped flow experiments were performed with a KinTek double syringe stopped flow accessory and a Horiba Fluoromax-4 spectrafluorometer (Edison, NJ, USA). Excitation was done at 490 nm and the donor emission was monitored at 520 nm. Each kinetic trace consisted of a thousand points and was measured 4–5 times at each IHF concentration. Each trace was fit to a single exponential using OriginPro. The experiments were done in IHF binding buffer that included 0.01% Nonidet P-40 instead of 10% ethylene glycol or glycerol. Association profiles were obtained by mixing the protein and DNA with a constant concentration of DNA (10 nm) and increasing concentrations of IHF (100 nM to 1500 nM) at 20 °C to allow kinetic analysis using a pseudo-first order binding model. Dissociation kinetics were measured by mixing a large excess of unlabeled junction (500 nM) with a fully bound labeled protein-junction complex and observing the loss of signal.

## 5. Conclusions

In summary, IHF binds to Holliday junctions with high affinity and a 1:1 protein: DNA ratio. Measurements with junction constructs containing shorter arms and the consensus sequence suggest that IHF binds to the junction center. Upon binding, IHF stabilizes the junction in the open conformation and shifts the population distribution to that state although the junction remains dynamic, suggesting formation of a partially dissociated state. This flexible, dynamic IHF-bound junction potentially facilitates the binding of other junction-binding proteins, such as resolvases. In contrast, IHF structural homolog HU binds to four-way junctions in a 2:1 ratio and stabilizes the stacked conformation [15]. Given that these proteins are abundant in the cell [64] and interact with the junction in contrasting ways, the competition between IHF and HU binding suggests that a delicate balance between junction opening and stacking might be mediated by these proteins. By inducing formation of a dynamic open conformation, IHF possibly facilitates branch migration while the stacked isoform induced by HU maybe required for junction resolution. Thus, the interplay between HU and IHF binding may play a role in regulating junction migration and resolution.

## Figures and Tables

**Table 1:** Time-Resolved Fluorescence Decay Parameters for the JH1 junction ^1^.

	α_1_ ^2^	τ_1_ (ns)	α_2_ ^2^	τ_2_ (ns)	α_3_ ^2^	τ_3_ (ns)	<τ> ^3^
JH1 donor only	0.45	0.27	0.44	2.37	0.11	5.79	1.81
JH1 donor-acceptor	0.46	0.11	0.23	1.00	0.31	3.72	1.43
With IHF							
JH1 donor only	0.44	0.27	0.38	2.07	0.18	5.02	1.79
JH1 donor-acceptor	0.43	0.22	0.32	1.64	0.25	4.44	1.72

^1^ Junctions were labeled in the following manner: JX-FAM and JR-TAMRA. In this scheme the iso II conformation has the highest energy transfer. ^2^ Relative amplitudes are reported, $\alpha_{i}=\frac{\alpha_{i}}{\sum_{i}^{}\alpha_{i}}$. ^3^ <τ> is the intensity-weighted lifetime defined as $\langle \tau \rangle =\frac{\sum_{i}^{}\alpha_{i}\tau_{i}}{\sum_{i}^{}\alpha_{i}}$ [50].

**Table 2:** Time-Resolved Fluorescence Decay Parameters for the J20 junction with and without IHF.

	α_1_ ^1^	τ_1_ (ns)	α_2_ ^1^	τ_2_ (ns)	α_3_ ^1^	τ_3_ (ns)	<τ> ^2^	E ^3^
J20 only								
Donor only	0.28	0.37	0.34	2.06	0.37	4.47	2.47	
Donor- Acceptor (XR)	0.41	0.4	0.38	1.56	0.21	4.00	1.58	0.36
Donor- Acceptor (XB)	0.26	0.28	0.28	1.50	0.46	3.50	2.13	0.14
J20 with IHF								
Donor only	0.30	0.40	0.35	2.07	0.35	4.49	2.44	
Donor- Acceptor (XR)	0.29	0.53	0.45	1.69	0.26	3.98	1.88	0.23
Donor- Acceptor (XB)	0.39	0.44	0.38	1.91	0.24	4.06	1.73	0.29

^1^ Relative amplitudes are reported; $\alpha_{i}=\frac{\alpha_{i}}{\sum_{i}^{}\alpha_{i}}$. ^2^ <τ> is the intensity-weighted lifetime defined as $\langle \tau \rangle =\frac{\sum_{i}^{}\alpha_{i}\tau_{i}}{\sum_{i}^{}\alpha_{i}}$ [50]. ^3^ Transfer efficiencies are calculated from the amplitude-weighted lifetimes using the following relationship $E=1-\frac{\tau_{D}}{\tau_{DA}}$ [50].

## Data Availability

Experimental data sets are available upon request to the corresponding author.

## Supplementary Materials

The following are available online at https://www.mdpi.com/article/10.3390/ijms24010580/s1.
