# Supplementary material for: Integration Host Factor Binds DNA Holliday Junctions
Source: Int J Mol Sci. 2022 Dec 29;24(1):580. doi: 10.3390/ijms24010580 (PMC9820253; doi:10.3390/ijms24010580)
Supplement: Supplementary file 1 [file ijms-24-00580-s001.zip › ijms-2068579-SI.pdf]

Integration Host Factor Binds DNA Holliday Junctions

Shawn H. Lin [1,2], Dacheng Zhao [1,2], Vivian Deng [1], Veronica K. Birdsall [1], Suzanne Ho [1], Olga Buzovetsky [1], Candice M. Etson [1,2] and Ishita Mukerji [1,2*]

Supplementary Materials

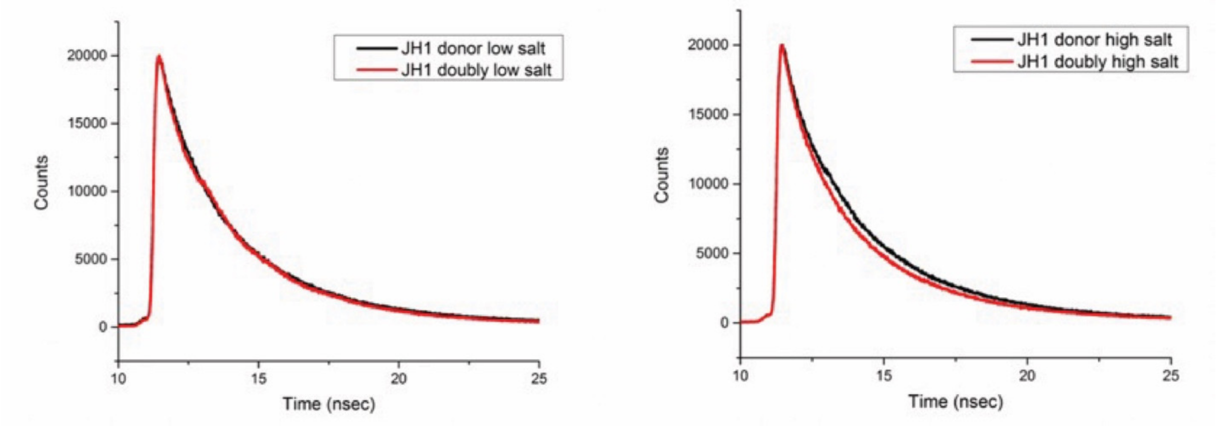

Figure S1: Time-resolved fluorescence decay measurements of the JH1 junction end-labeled with either FAM only (donor) or FAM and TAMRA (doubly). The faster decay of the JH1 doubly-labeled junction in high salt is indicated of a stacked junction. Experimental conditions as described in the Materials and Methods.

Table S1: Time-resolved fluorescence decay parameters JH1 junction

	DNA	α_1 [1]	τ_1 (ns)	α_2 [1]	τ_2 (ns)	α_3 [1]	τ_3 (ns)	<τ> [2] (ns)
Low salt	JH1 X-FAM	0.35	0.627	0.51	2.67	0.13	5.04	2.23
	JH1 X-FAM, R-TAMRA	0.74	1.03	0.07	4.31	0.18	2.45	2.31
High salt	JH1 X-FAM	0.44	0.27	0.44	2.37	0.11	5.79	1.81
	JH1 X-FAM, R-TAMRA	0.46	0.11	0.23	1.00	0.31	3.72	1.43

[1] Relative amplitudes are reported, $\alpha_i = \frac{\alpha_i}{\sum_i \alpha_i}$. [2] <τ> is the intensity-weighted lifetime defined as $\langle\tau\rangle = \frac{\sum_i \alpha_i\tau_i}{\sum_i \alpha_i}$).

Table S1: Decay parameters for the JH1 junction under high (70 mM KCl) and low salt (10 mM KCl) conditions.

JH1 X-FAM_R-TAMRA High salt	∝	Weighted-∝	% of Populations	
Component #1	19837	0.46	69%	**% *Iso II* Population exhibiting FRET**
Component #2	10102	0.23		
Component #3	13333	0.31	31%	**% *Iso I* Population exhibiting No FRET**

Table S2: Population analysis of JH1 junction from decay parameters (Table S1)

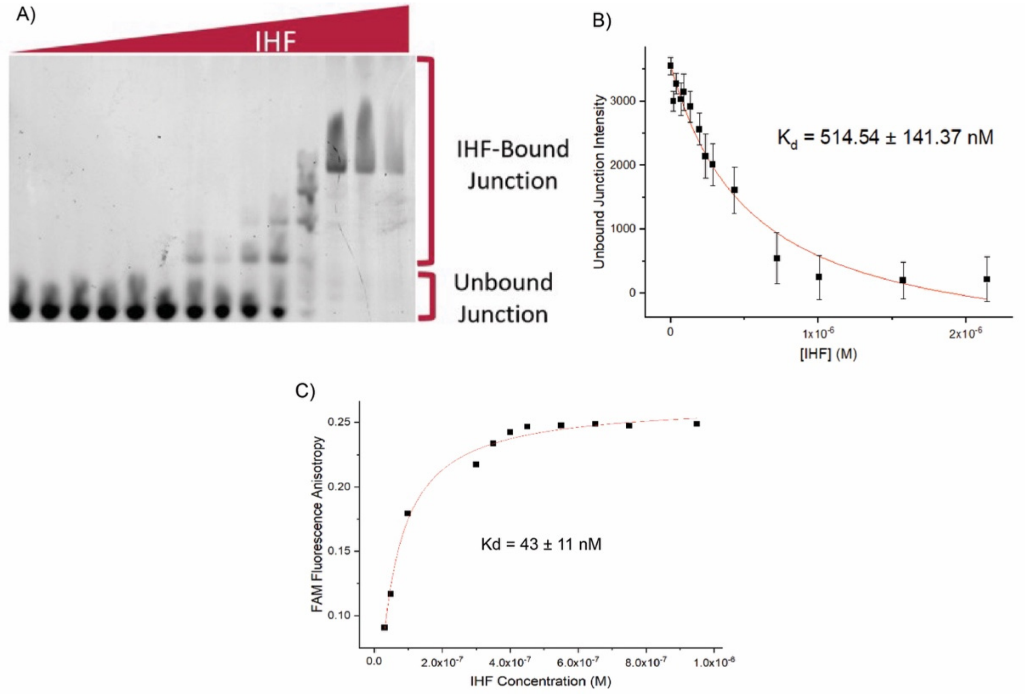

Figure S2. A) EMSA of IHF binding to J20 (5 nM) in 1mM Mg^{2+}. B) Analysis of free DNA yields a $K_{D(app)}$ in gel of 514 +/- 141 nM. C) Fluorescence anisotropy measurements yield a K_D of 43 ± 11 nM. Experiments were performed and analyzed as described in the text. As discussed in the main text, the much larger dissociation constant in the gel is potentially attributed to dilution effects in the gel and also because of conformational transitions of the junction that lead to slower migrating species.

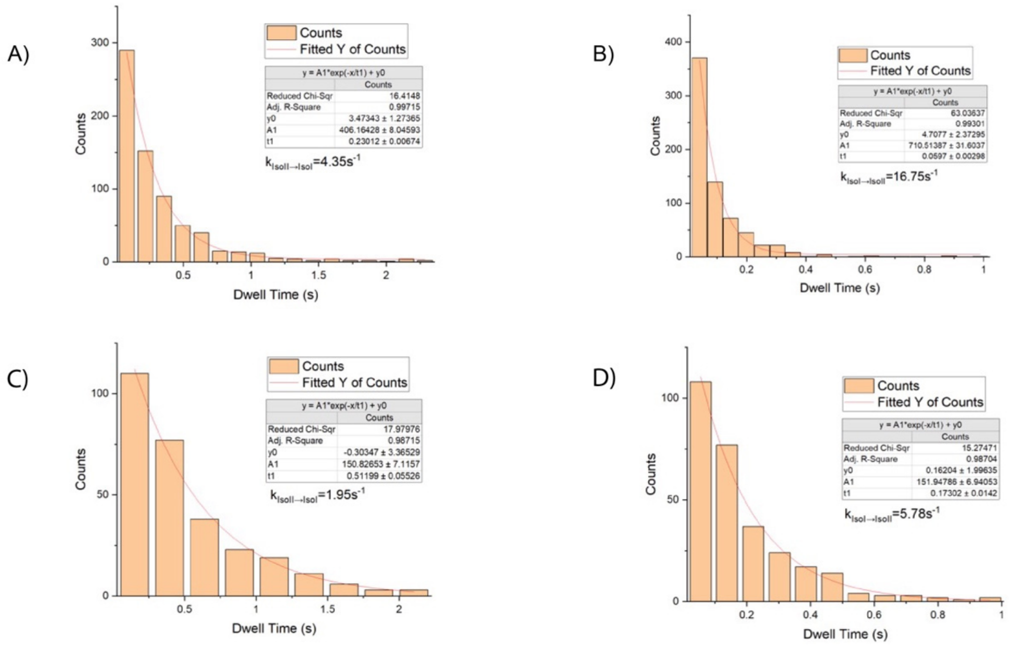

Figure S3. Dwell time analysis of XR labeled J20 junctions in 50 mM Mg^{2+} (A & B) and 10 mM Mg^{2+} (C & D). A) Dwell time distribution of high FRET(~0.8) or iso II state for XR labeling scheme in 50 mM Mg^{2+}. B) Dwell time distribution of low FRET (~0.2) or iso I state for XR labeling scheme in 50 mM Mg^{2+}. C) Dwell time distribution of high FRET(~0.8) or iso II state for XR labeling scheme in 10 mM Mg^{2+}. D) Dwell time distributions of low FRET(~0.2) or iso I state for XR labeling scheme in 10 mM Mg^{2+}.

Transitions between the high and low FRET states were identified using the vbFRET algorithm in MATLAB, and the lifetime of each state was extracted from the histogram of dwell times for that state by fitting to a single exponential decay. The transition rate out of each state is the inverse of its lifetime. In both cases, the lifetime of the iso II state was longer than the lifetime of the iso I state, reflecting the dominance of the iso II population in the single molecule FRET efficiency histograms. For both states, the transition rates were slower in the presence of 10 mM Mg^{2+} than for 50 mM Mg^{2+}.

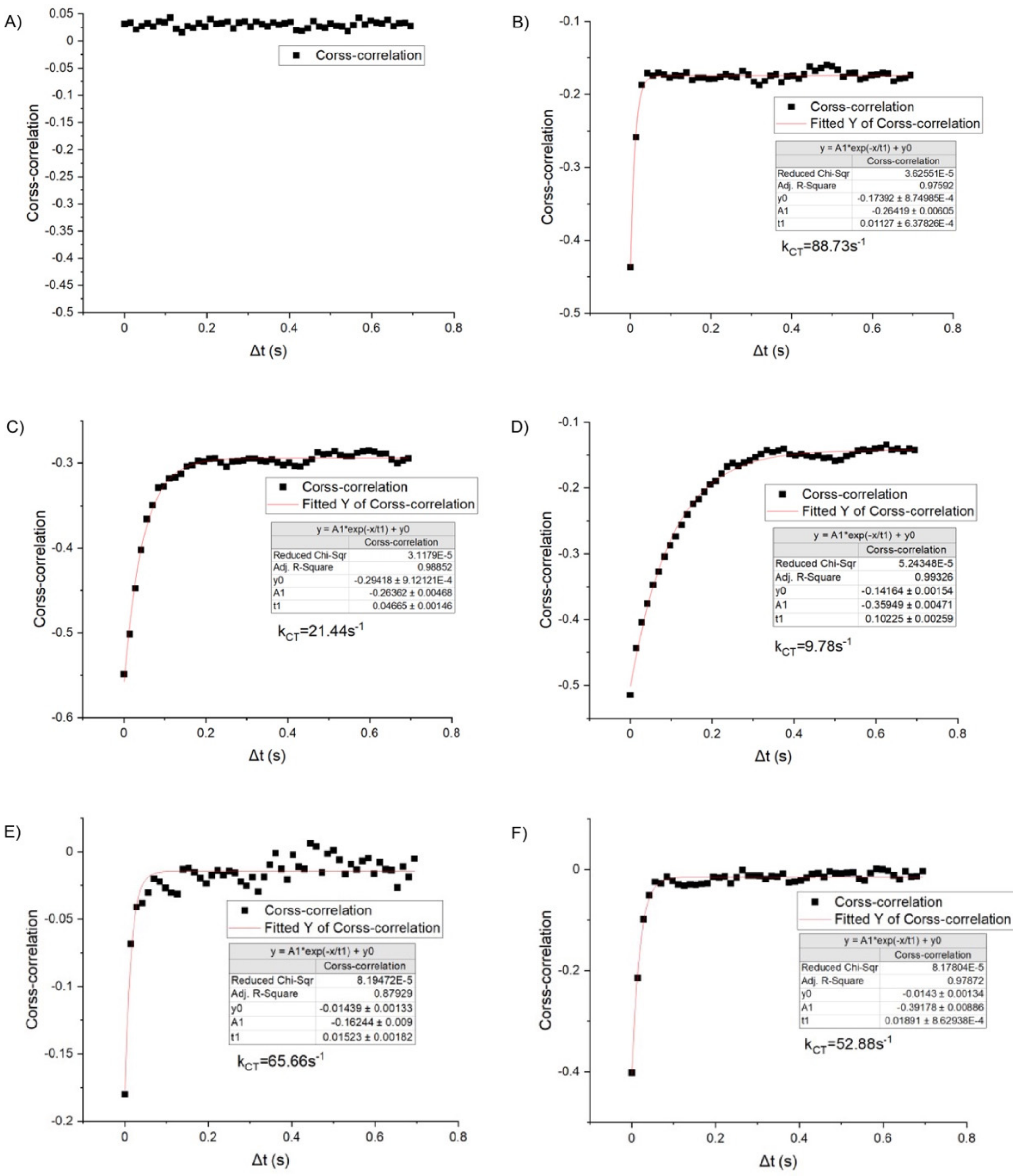

Figure S4. Cross correlation analysis of Holliday Junctions in different imaging buffers. All measurements were carried out in the imaging buffer with the following additions: A) 1mM EDTA, B) 1mM Mg^{2+}, C) 10mM Mg^{2+}, D) 50mM Mg^{2+} E) 1 mM Mg^{2+} with IHF F) 10 mM Mg^{2+} with IHF. All data were well-described with a single exponential function as shown. The conformer transition rate (k_{CT}) was obtained from the lifetime determined from the fit.

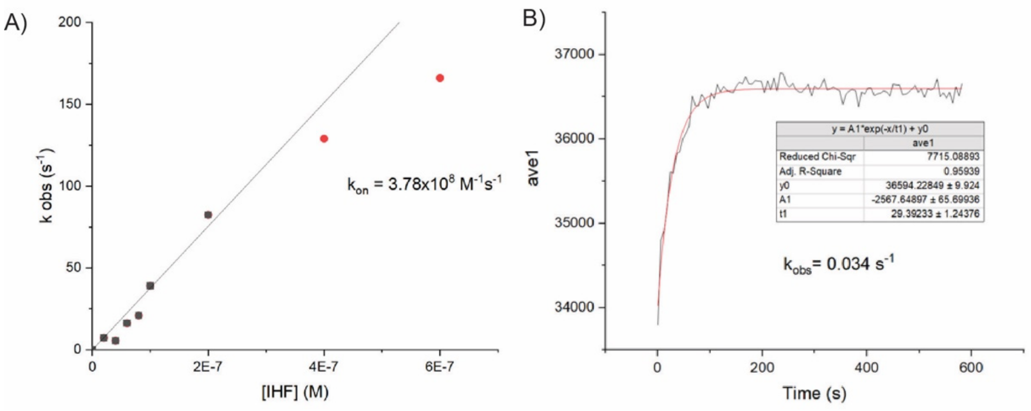

Figure S5. Stopped-flow FRET of IHF binding to 34 bp duplex containing the H1 consensus sequence. A) Plot of measured k_{obs} as a function of IHF concentration, yielding a k_{on} rate of 3.78 x 10^8 $M^{-1}s^{-1}$. DNA concentration was maintained at 10 nM and IHF concentration was increased from 50 nM to 600 nM. Measurements were done with an excess of protein to generate pseudo first order reaction conditions. B): Representative kinetic trace of the binding reaction which is well described by a first-order exponential as shown.
